# Pandemic preparedness and response: a survey among experts from high- and low-middle-income countries about the “100 Days Mission”

**DOI:** 10.3389/fpubh.2025.1617066

**Published:** 2025-09-05

**Authors:** Vanessa Pencelli, Ralf Clemens, Mihai Alexandru Bica, Sue Ann Costa Clemens

**Affiliations:** ^1^Institute of Global Health, University of Siena, Siena, Italy; ^2^Department of Pediatrics, Oxford University, Oxford, United Kingdom

**Keywords:** pandemic preparedness, 100 Days Mission, CEPI, high income countries, low middle income countries, survey

## Abstract

**Background:**

The “100 Days Mission” (100DM), designed by the Coalition for Epidemic Preparedness Innovations (CEPI), represents an ambitious new concept in vaccine development for effective pandemic preparedness, rapid response, and the reduction of health inequalities. We aimed to identify potential obstacles to the success of the 100DM by conducting a survey among experts in vaccinology and public health from both high-income countries (HICs) and low- and middle-income countries (LMICs).

**Materials and methods:**

A descriptive cross-sectional study was conducted, using a semi-structured online survey distributed to 116 experts from both LMICs and HICs. The data collected were analyzed quantitatively and qualitatively, highlighting the differences in responses between the LMICs and HICs respondents.

**Results:**

The overall response rate was 73.2% (85/116), with 74% (57/77) of the respondents from HICs and 69.2% (27/39) from LMICs. The LMIC respondents (14/27, 51.9%) were more confident in the success of the 100DM than the HIC respondents (15/57, 26.3%). Additionally, LMIC respondents believed more strongly in the potential impact to overcome inequalities (15/27, 55.6% vs. 19/57, 33.3%). Almost all experts from both LMICs and HICs considered political will and governance, and sufficient sustainable funding as the most important prerequisites for the success of the 100DM, followed by the need for trustful collaborations between HICs and LMICs, effective public–private partnership, and continuous training and capability building. The 100DM should prioritize the establishment of vaccine candidate libraries, enhancement and sustainability of surveillance capabilities, and creation of laboratory and clinical trial site networks.

**Conclusion:**

This is the first prospective survey evaluating the feasibility of 100DM, involving external stakeholders from both HICs and LMICs. Experts from LMICs are more confident in the success of the 100DM than those from HICs. Political will, good governance, and sustainable financing are essential for successful implementation. The technical innovation aspect of the 100DM should prioritize the development of prototype vaccines and operational components over more long-term initiatives with broader impacts. To realize CEPI’s vision, global stakeholders must set priorities and commit to focused, coordinated actions. Achieving early wins through short-term, high-impact deliverables and actionable policy reforms is essential to build confidence, sustain momentum, and prevent stakeholder fatigue. This strategic prioritization underpins the initiative’s long-term success.

## 1 Introduction

In 2024, there were 17 outbreaks of “dangerous diseases,” including recent cases of the Marburg virus, Mpox, and H5N1 avian influenza, highlighting the ongoing risks to society [as noted in the World Health Organization (WHO) comment on the 2024 Global Preparedness Monitoring Board report] (1). A recent risk modeling indicated that there is a 27.5% chance of a new pandemic as devasting as COVID-19 occurring in the next 10 years; however, if effective vaccines are rolled out within 100 days after the discovery of a new pathogen, the likelihood of a pandemic drops from 27.5 to 8.1% (2).

The impact of vaccines was once again demonstrated during the COVID-19 pandemic. Once vaccines became available, they dramatically changed the course of the pandemic, saving over 14 million lives worldwide in just the first year of their availability (3).

The first COVID-19 vaccines were developed and approved within 326 days of genome sequencing, making an unprecedented achievement compared to traditional timelines (4, 5). However, even this accelerated timeline was insufficient to prevent millions of deaths and huge socioeconomic costs. In response, the Coalition for Epidemic Preparedness Innovations (CEPI), endorsed by the G7, proposed a new strategy for even faster vaccine development along with equitable access during future pandemics—referred to as the “100 Days Mission” (100DM) (6–9). This paradigm shift in the current development processes involves activities that need to be undertaken as preparedness measures during the pre-pandemic period in order to be ready for a global and equitable vaccine response within 100 days of identifying a new pathogen with pandemic potential. The preparedness plan includes establishing libraries of vaccine prototypes, global clinical trials and laboratory networks, global surveillance systems, global biomanufacturing capacity, and early biological markers (10–12).

It is estimated that, in conjunction with the history of lifting non-pharmaceutical interventions, the 100DM could have averted 8.33 million deaths [95% credible interval (CrI) 7.70–8.68] during the first year of the COVID-19 pandemic, resulting in monetary savings of US$14.35 trillion (95% CrI 12.96–17.87) (13).

Concerns have been raised regarding the realism and likelihood of success of the 100DM. This study aimed to collect insights from public health and vaccine development experts in both high-income countries (HICs) and low- and middle-income countries (LMICs) on the feasibility of the Mission, as well as to identify critical success factors and obstacles for the 100DM.

## 2 Materials and methods

### 2.1 Study design

This was an online survey that used a semi-structured questionnaire to gather information from experts in both HICs and LMICs, conducted between 25 September and 11 November 2023.

### 2.2 Survey population

The survey attempted to include 120 opinion leaders from LMICs and HICs with specific expertise across the vaccine development process, including areas such as research, chemistry, manufacturing, and controls (CMC), manufacturing processes, clinical research and development (R&D), regulatory affairs, supranational/national governmental and non-governmental organizations (NGOs). It also sought inputs from policy makers, such as members of National Immunization Technical Advisory Groups—(NITAGs), and representatives from philanthropic organizations.

These experts were selected based on their participation in COVID-19 discussion forums and professional networks, their research and publications on pandemic preparedness, and their expertise in vaccine development.

### 2.3 Data collection

Data were collected through an online questionnaire sent to the experts through a link shared in an email invitation. This link also contained a detailed summary document of the 100DM as a reminder. Before completing the survey, each potential participant was required to provide voluntary consent to participate in the study and acknowledge the statement of confidentiality and anonymity.

The survey consisted of 12 questions, including both multiple-choice and open-ended formats (Supplementary material 1). The questionnaire was prepared and validated by subject matter experts from the CEPI and the Institute for Global Health, University of Siena, Italy, and internally tested for its feasibility, consistency, timing, and technical functionality. The survey addressed technical, operational, and governance aspects, including the following:

- Feasibility of the 100DM.
- Identification of responsible stakeholders to initiate the response reaction phase.
- Current state of pandemic preparedness, especially in LMICs.
- Likelihood of success, obstacles, and impact of the 100DM processes/innovations proposed by the CEPI.
- Evaluation of the 100DM as a tool to ensure equitable vaccine access.

### 2.4 Data analysis

Data were exported from the original collection platform, Jotform.com, to Microsoft Excel, where the collected information was stored, cleaned, and further analyzed.

Quantitative variables (such as the respondents’ profile, overall assessment of the 100DM, current state of pandemic preparedness, and ranking of 100DM processes) were expressed as proportions. Qualitative data were analyzed and reported descriptively. Repetitive themes during the qualitative analysis were coded and grouped into thematic categories.

The results were analyzed as a total and by subgroups of HICs and LMICs. Although no comparative statistics were performed, the outcomes are reported descriptively.

### 2.5 Ethical considerations

This survey did not include any intervention or the collection of personal information; therefore, no ethical approval was required. Data collection was conducted anonymously and confidentially, ensuring that respondents were not identifiable during the data analysis and reporting of results.

## 3 Results

**Figure 1:**
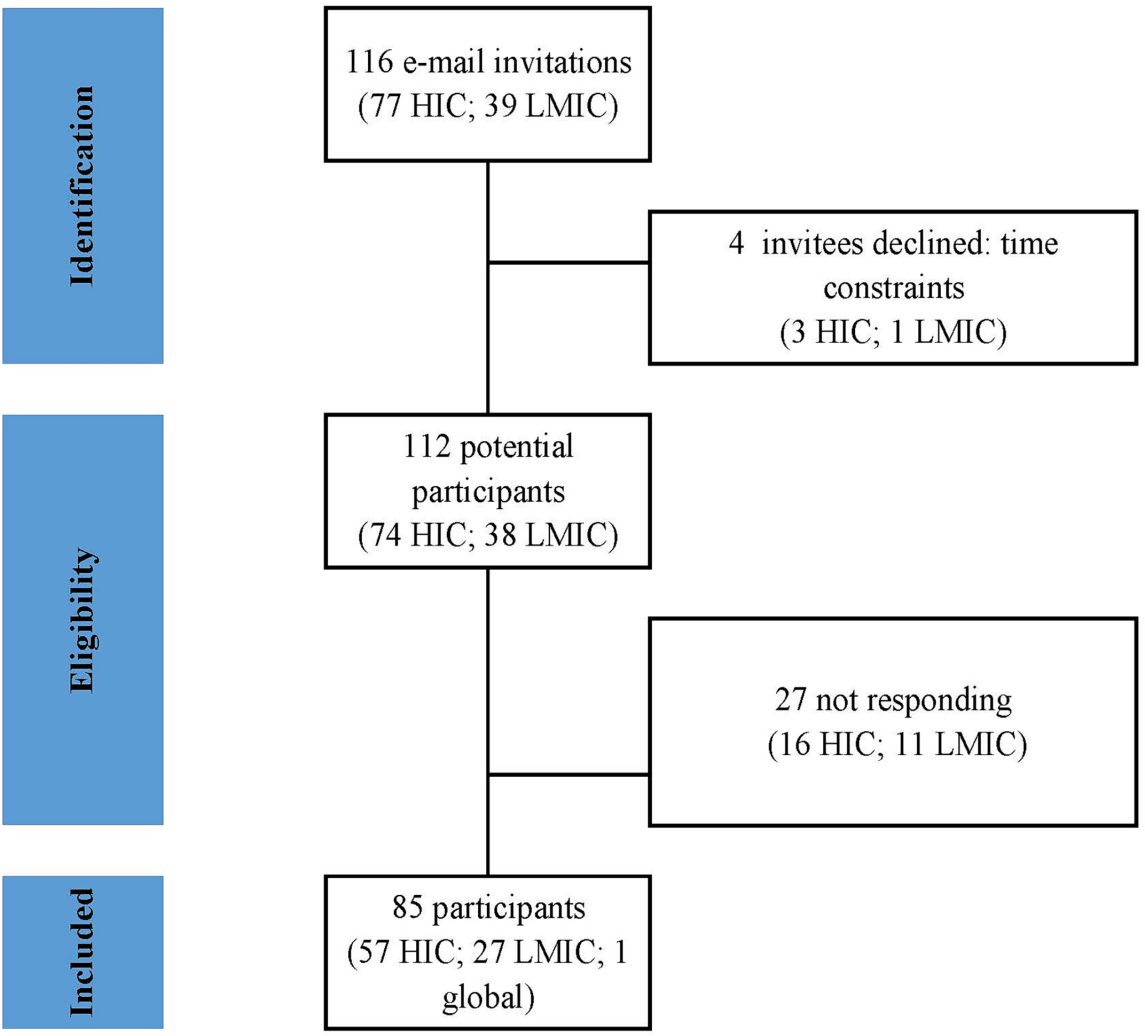
Flow diagram of study screening and inclusion.

A total of 116 experts were selected and invited by email to participate in the survey. Of these participants, 85 (73.2%) accepted the invitation, with 57/77 (74%) from HICs and 27/39 (69.2%) from LMICs. One participant declared himself “global” (Figure 1).

A total of 31/57 (54.3%) HIC experts were from Europe, 15 (26.3%) from North America, and 11 (19.2%) from the Asia-Pacific region (South Korea, Singapore, Japan, Oceania). Among the LMIC respondents, nearly half were from Africa (13/27, 48.1%), 11 (40.7%) from Latin America, and three (11.1%) from South/South-East Asia.

**Figure 2:**
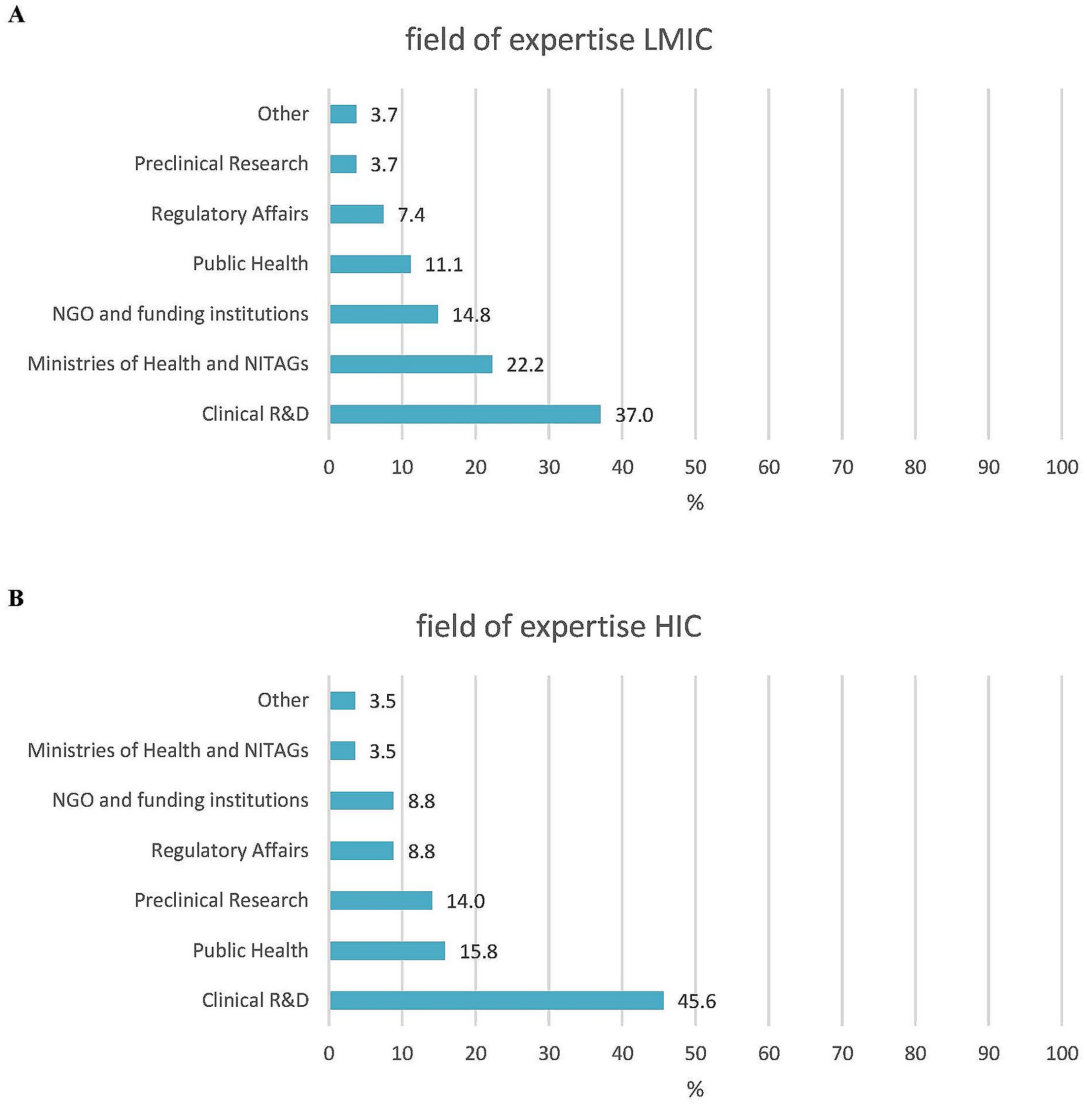
Fields of expertise. **(A)** LMIC experts (*N* = 27). **(B)** HIC experts (*N* = 57).

**Figure 3:**
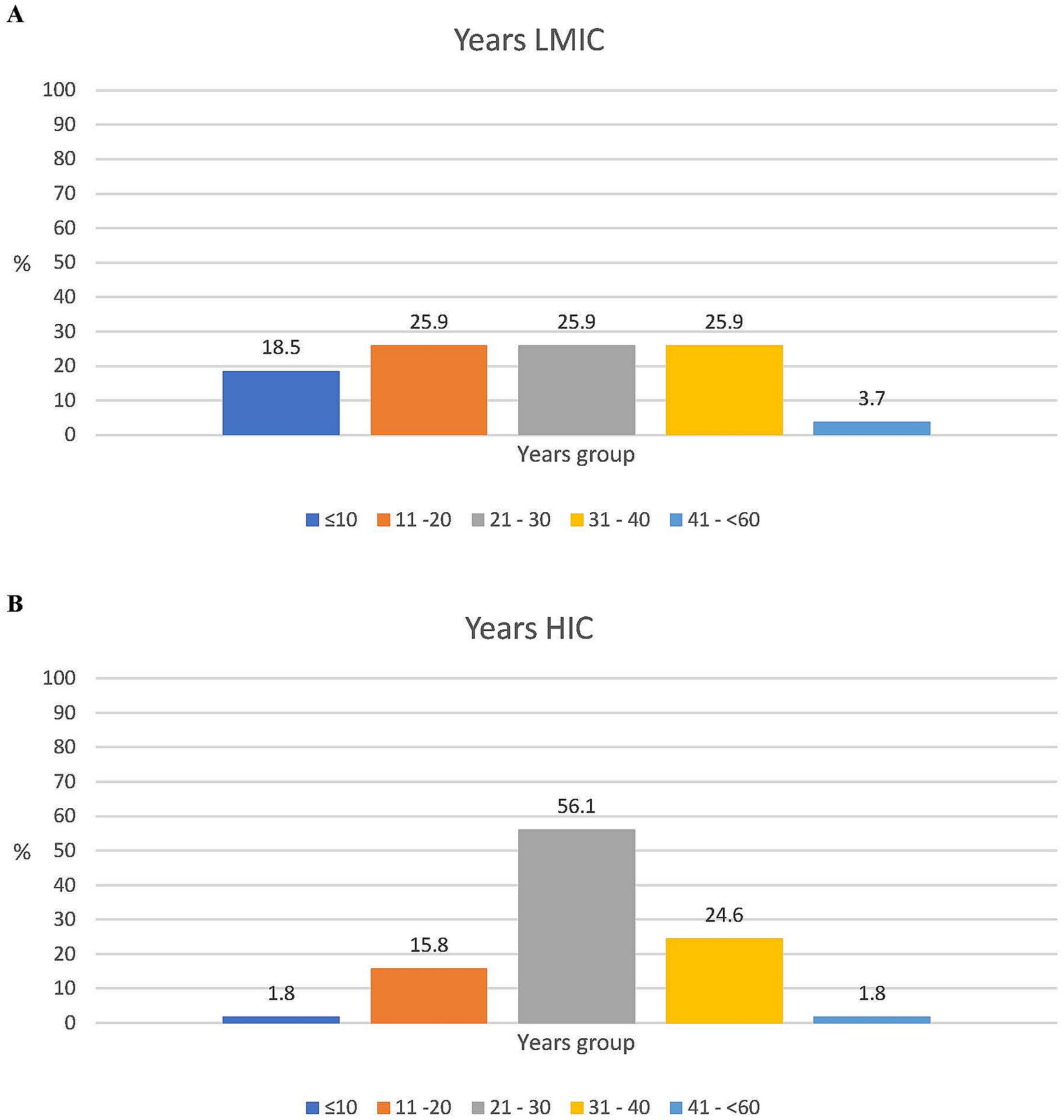
Years of expertise. **(A)** LMIC experts (*N* = 27). **(B)** HIC experts (*N* = 57).

In addition, 37/85 of the respondents (43.5%) were experts in clinical development, while 12 (14.1%) were in public health, and the remaining 42.8% represented expertise in vaccine research, NGOs, philanthropy, supranational/national policymaking, and regulatory affairs. There were more experts from LMICs than HICs with a governmental/NITAG background (6/27, 22.2% vs. 2/57, 3.5%, respectively). For all other areas of expertise, the difference between the LMIC and HIC respondents was less than 10% (Figure 2). A total of 63/85 (74.1%) respondents had more than 20 years of experience in vaccinology, with a median of 29 years (range 2–55 years). In general, the LMIC participants had less experience than the HIC participants: 44.4% (12/27) versus 17.5% (10/57) had 20 years or less of experience and 18.5% (5/27) versus 1.8% (1/57) had 10 years or less of expertise in their specific field, respectively (Figure 3).

When asked about their confidence in the feasibility and suitability of the 100DM to meet its goals, approximately half of the opinion leaders were skeptical (39/85, 45.9%) and 18.8% (16/85) had a neutral opinion, while one in three (29/85, 34.1%) believed in its success. Confidence in the success of the Mission was, however, different between the HIC and LMIC respondents. Among the LMIC experts, 51.9% (14/27) believed that the Mission would succeed, while 18.5% (5/27) stated that it would fail. In contrast, confidence among the HIC experts was much lower, with only 26.3% (15/57) believing in its success and 57.9% (33/57) anticipating failure. These skeptical respondents considered the 100DM too ambitious and unrealistic to be achieved within a reasonable timeframe, warning that failure to deliver on its promises could further weaken public trust. However, the believers praised the CEPI for its boldness in launching this initiative, describing it as “an ambitious project that motivates change and triggers a complete reassessment of how we develop and deploy vaccines” and as a tool that “helps build resilience, capability, and capacity.” The CEPI was also seen as a “guardian to remind society of pandemic risks and preparedness needs.”

To mitigate the risk of failure and miscommunication, the experts offered various recommendations:

- Clearer, cautious, and more widespread communication in lay terms directed at civil society about the 100DM, presented with realism to avoid a communication disaster and further erosion of trust in the event of failure.
- Clearer definition and coherent communication to policymakers and the scientific community regarding what constitutes Day Zero as the start of the clock. Suggestion criteria included the public availability of a genome sequence for a “pathogen with exponential growth, high potential of significant spread across a region, without vaccine availability,” [sic] as well as epidemiological indicators such as “evidence of continuous human-to-human transmission,” “zoonotic infection with community outbreaks,” and “uncontrollable outbreak with >1% mortality.”
- As phase III/efficacy data cannot be available within 100 days, stakeholders should agree on a minimum clinical data package before vaccine rollout to avoid further trust issues.
- The declaration of a pandemic was considered by the majority of the respondents to be a prerogative of the WHO; however, over 50% also commented that this decision should not rest solely with the WHO but include the CEPI, regional/local governance bodies, external scientific experts, and vaccine manufacturers. A small group of the LMIC and HIC respondents proposed the creation of a specific global task force/pandemic response organization “with a centralized management as a strong health authority,” supported by the WHO but including regional representatives, as well as scientists and manufacturers from both HICs and LMICs. This dedicated consortium should act independently and impartially, with the sole goal of developing a global pandemic strategy and implementing resolutions.
- The CEPI, rather than the WHO, should be the facilitator to ensure alignment among various stakeholders (“preclinical scientists, clinicians, regulatory bodies, policymakers, supranational agencies, governments, funders, and manufacturers”).
- Develop strategies and allocate funds to address vaccine hesitancy and misinformation proactively.
- As most outbreaks originate in LMICs, surveillance, laboratory, and clinical development capacities need to be significantly enhanced in these regions. This includes establishing and maintaining pre-qualified trial sites with monitoring capacities for large-scale studies, eventually coordinated by non-profit organizations.
- Pharmacovigilance aspects need to be addressed in the 100DM.

**Figure 4:**
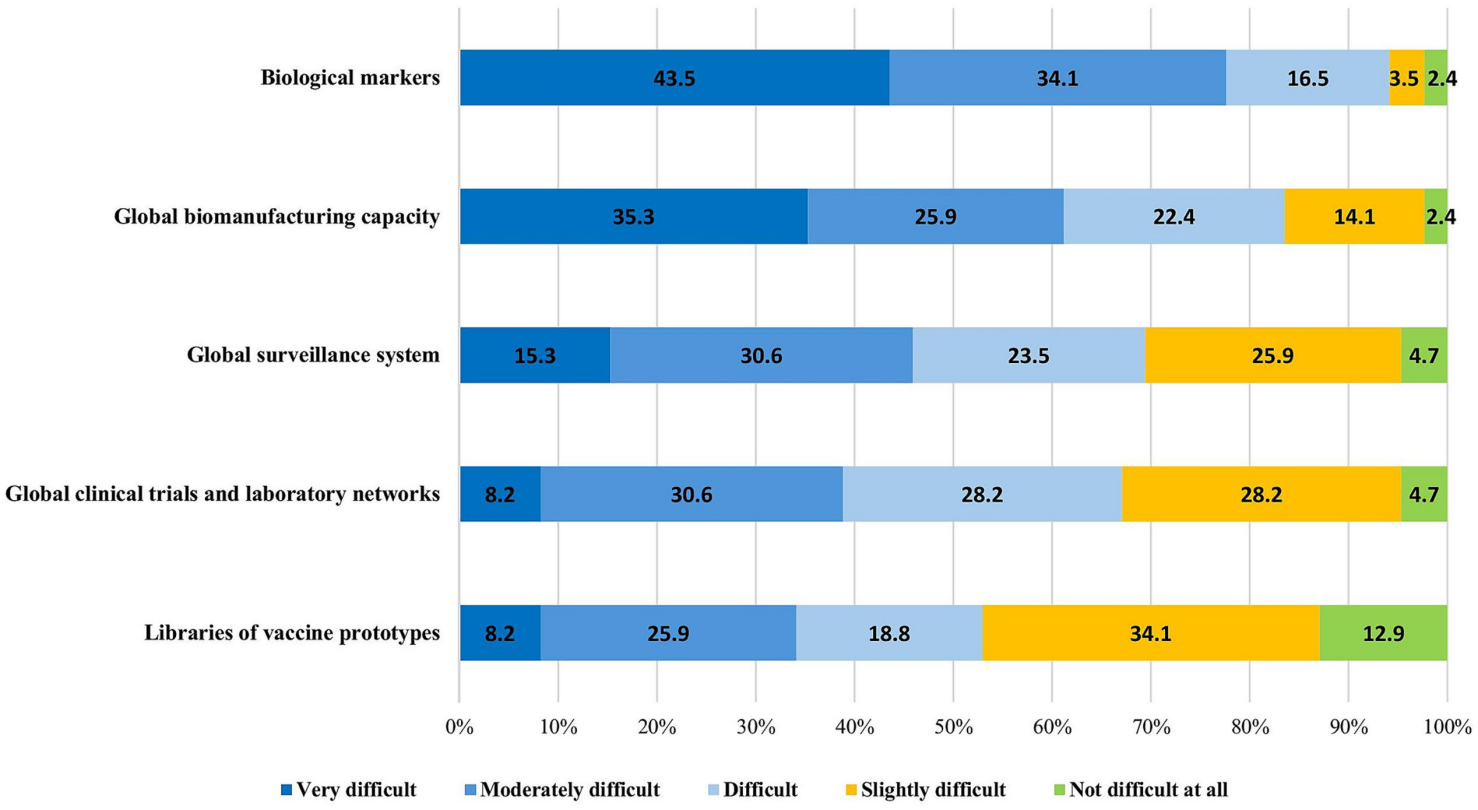
Difficulties in the implementation of the CEPI’s pre-pandemic preparedness technical innovations.

The experts were asked to identify technical and managerial challenges to the implementation of the CEPI’s five innovation areas: global surveillance systems, global manufacturing capacity, early biological markers, global clinical trials and laboratory networks, and libraries of vaccine prototypes. There was general concordance between the LMIC and HIC respondents regarding the difficulties in the implementation of these innovations. The majority of the participants (80/85, 94.1%) stated that identifying early biological markers as correlates of protection within 100 days is a difficult, if not impossible, task and should be deprioritized. Similarly, the creation of a sustainable global biomanufacturing capacity—while critical to ensuring equal access—was seen as difficult to achieve by 71/85 (83.5%) respondents, mainly because of the risk of insufficient and inconsistent funding, as well as the risk of insufficient utilization of the plants during interpandemic cycles. In contrast, between 31 and 47% of the respondents considered establishing a global surveillance system, clinical trial and laboratory networks, and prototype libraries to be technically feasible (Figure 4).

Over 75% of the respondents noted that surveillance systems, clinical/laboratory networks, and especially manufacturing facilities are set up for failure if they are not kept “warm” through continuous allocation of work, ensuring they remain fully operational when a pandemic strikes.

**Figure 5:**
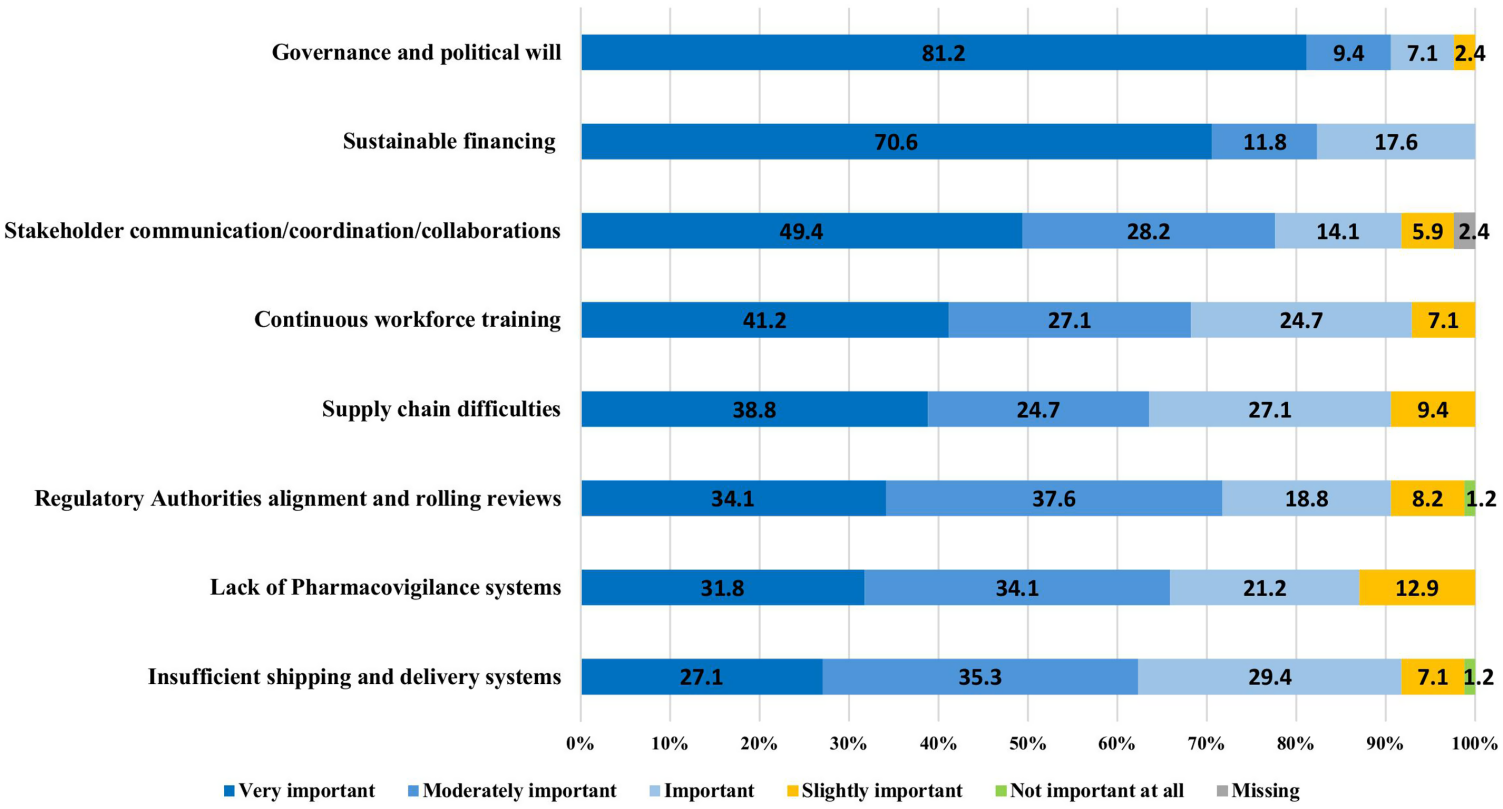
Implementation challenges of the 100DM.

The experts from LMICs and HICs were aligned on the implementation and operational challenges of the 100DM (Table 1):

- All 85 respondents showed concerns about the sufficient and sustainable financing of pre-pandemic efforts, and 2/3 considered it as the most critical obstacle to avoid failure (Figure 5). Insufficient and unstable financing would mostly impact the development of global biomanufacturing capacity, global clinical trial and laboratory networks, and global surveillance systems (Table 1).
- Governance and political will: over 90% (83/85) of the respondents (Figure 5) highlighted that the Mission is bound to fail without clear governance rules and political will from both HIC and LMIC stakeholders. Specific concerns raised included nationalism in vaccine supply, limited specimen sharing, lack of benefit-sharing, denial of pandemic threats by governments, unwillingness to invest beyond election cycles, and lack of clarity regarding the guardian of measures during a pandemic.
- Communication, coordination, and collaboration between stakeholders—particularly between countries (especially North–South), between countries and the industry, within the industry itself, and between the industry and the WHO. Root causes included ownership issues; intellectual property (IP); lack of trust between non-governmental organizations, governmental organizations, and the industry; and denial to accept that competencies might lie with other stakeholders. This lack of stakeholder collaboration would primarily affect global clinical trials and laboratory networks, surveillance systems, and vaccine libraries (Table 1).
- Other main areas to address include training and capabilities building across the vaccine development and access chain, as well as regulatory alignment through mutual reliance and rolling reviews (Figure 5).

**Table 1:** Main technical challenges to the 100DM innovations.

Innovation	Technical challenges	
Biological markers	Lack of knowledge and proof of concept (50%) Technical issues and harmonization (24%) Regulatory buy-in (8%)	Financing (6%) Collaboration and cooperation (6%) Capabilities and capacity building (5%) Political will (1%)
Global biomanufacturing capacity	Financing (36%) Sustainability (18%) Manufacturers’ collaboration and cooperation (17%)	Capabilities building (16%) Political will (8%) Regulatory capacity (2.5%) Needs for advanced technology and logistics (2.5%)
Global disease surveillance system	Collaboration, coordination, and mutual interests (30%) Financing (27%) Capacities and capabilities building (14%)	Political issues (12.5%) Sustainability issues (11%) Regulatory issues (3.5%) Community engagement (2%)
Global clinical trial and laboratory network	Collaboration and coordination between sites, funders, and developers (36.5%) Sustainable financing (30.5%) Harmonization of clinical trials and regulatory procedures (12%)	Continuous training (9%) Political will (7%) No challenges (5%)
Libraries of vaccine prototypes	Industry’s coordination and collaboration (25%) Pathogen-related issues (22%) Financing (21%)	Prototype efficacy and choice of adequate platform (17%) Continuous training (7%) No challenges (6%) Political will (2%)

The objective of the 100DM to overcome access inequality during a pandemic was judged differently: over half (55.6%, 15/27) of the LMIC respondents believed that this project would be impactful in achieving access equality, while 63.2% (36/57) of the HIC experts believed it would not have this impact, due to lack of political willingness, vaccine nationalism, lack of funding, industry greed, and insufficiently trained human resources.

A core strategy for achieving access equality is “geodiversifying” manufacturing to LMICs through staged technology transfers. However, this is not a quick win and requires careful management of expectations. Secondly, capacity building in clinical development, regulatory sciences, and pharmacovigilance was also considered a key priority.

Despite differences in opinions about the 100DM’s impact on vaccine equity, there was an agreement between the two groups regarding the current level of pandemic preparedness of LMICs compared to the situation prior to the COVID-19 pandemic. The majority of the respondents from LMICs (81.5%, 22/27) believed their region is better prepared for the next pandemic, with a similar perception among the HIC respondents (59.6%, 34/57).

## 4 Discussion

To the best of our knowledge, this is the first external survey about the feasibility of the 100DM involving experts in vaccine development and public health from HICs and LMICs.

The 100DM is an ambitious paradigm shift in how vaccines are developed and made broadly available, with the aim to reduce the development timeline from the traditional five to 10 years to 100 days, approximately one-third of the time it took for the first COVID-19 emergency vaccine authorization, which was itself unprecedented.

More experts from HICs were skeptical about the Mission’s feasibility compared to the cautious optimism shown by the LMIC group (26.3% vs. 51.9% of believers, respectively). Similarly, the LMICs experts had more confidence than their HICs peers in the Mission as a tool to overcome inequalities. This last aspect is fundamental for pandemic prevention: it has been noted that, although there are multitudes of reasons for the (re)emergence of infectious diseases and outbreaks, the disease burden is fundamentally determined by social and health inequities, especially in LMICs (14). This lack of confidence in the success of the 100DM among HICs experts is problematic and needs to be addressed, as they should serve as key advocates of the Mission to political and other crucial stakeholders to ensure sustainability.

For many of the study participants, the definition of Day Zero was unclear. The guardians of the 100DM need to define it and communicate it more clearly to both the scientific community and civil society, setting clear expectations for the Mission.

The experts differed on what should trigger the initiation of the “reaction phase,” with suggestions ranging from the genome sequence availability of an outbreak pathogen to epidemiological data showing zoonotic or human-to-human transmission. Although criteria might differ by pathogen, they should be clearly established and communicated.

There was consensus that the WHO should play the leading role in declaring a potential pandemic and initiating Day Zero; however, this should not be done in isolation but in collaboration with other competent stakeholders.

The 100DM is divided into five workstreams that, according to the survey, have very different likelihoods of success and different immediate or long-term impacts. Early identification of biomarkers or correlates of protection in humans, as well as establishing a global manufacturing infrastructure, was considered the most difficult to achieve rapidly. The survey participants recommended that, before engaging in technology transfers or investing in greenfield facilities, long-term sustainability needs to be ensured to avoid the failure of various past transfer attempts. Upfront investments need to include a long-term commitment to capacity building beyond the manufacturing facility. It needs to be clear to the involved stakeholders that parallel investment in clinical, regulatory, and pharmacovigilance capabilities is mandatory. Competitive business interest, lack of incentives for technology transfers and for collaboration between manufacturers, and uncertainties around IP were identified as other major obstacles. Incentives for pathogen sharing during an outbreak, such as binding equity agreements, were proposed as a potential solution, aligned with recent public recommendations (15).

The experts also requested clarification on whether the manufacturing “geodistribution” was meant solely for pandemic preparedness or if it would extend to routine vaccines as well. In that context, it was recommended not to focus on just one platform such as mRNA, which has proven to be well-suited for pandemics but lacks comparable evidence of performance against other vaccine-preventable diseases when compared to other platforms, such as virus-like particles. Furthermore, it needs to be ensured that not only manufacturing but also clinical sites are kept “warm” during interpandemic cycles to avoid losing capabilities. Keeping mRNA facilities “warm” between outbreaks could be challenging, given the almost complete lack of other mRNA licensures. To ensure the sustainability of a regionalized manufacturing capability, improving the regulatory maturity levels of the National Regulatory Agencies and building pharmacovigilance expertise are of equal critical importance in those countries.

Successful examples of technology transfers during the pandemic that should serve as sources of learning include the partnerships between Oxford University, AstraZeneca, the Serum Institute of India, and Fiocruz/Biomanguinhos for the Chad–Ox COVID-19 vaccine (16, 17). Another example is the COVID-19 vaccine project developed by Texas Children’s Hospital Center for Vaccine Development at Baylor College of Medicine, now produced by LMIC manufacturers in India and Indonesia (18). All development processes were published with open access, and no IP restrictions were imposed on the licensors.

Most diseases listed in the previous WHO Blueprint are expected to emerge in LMICs (19). In addition to manufacturing, laboratory and clinical trial infrastructures are probably the most imbalanced between HICs and LMICs. These gaps need to be rectified in the pre-pandemic preparation phase, addressed in a coordinated way, and led by a few organizations, selected based on competence rather than political considerations, as per experts’ recommendations.

Clinical trial sites in LMICs need to be kept “warm,” with a constant flow of studies during interpandemic periods to ensure immediate readiness during an outbreak. The expert participants recommended that multinational vaccine manufacturers, as well as those from LMICs, be required to execute part of their clinical development plans in LMICs to ensure site readiness for a pandemic.

Concrete proposals for building regulatory capacity included establishing collaborative medicines registration networks, such as ZAZIBONA in Southern Africa (20), embracing the concept of “reliance,” and implementing rolling submissions as a routine procedure, which, of course, would have staffing consequences.

Reinforcing surveillance systems is not seen as a major technical hurdle, as it can build on established mechanisms such as the Global Influenza Surveillance and Response System (GISRS) and arbovirus surveillance networks (21, 22). However, ensuring participation from all regions might be problematic due to differences in political, legal, and diplomatic frameworks, as well as concerns about stigmatization and potential impacts on tourism and industry.

Unanimously, the survey participants were deeply concerned about the implementation challenges of the 100DM concept, especially during the preparedness phase. Sustainable and sufficient financing was highlighted by every participant as the greatest obstacle to success, as is the case with any pandemic preparedness initiative (23). At-risk financing during interpandemic periods, mainly with respect to manufacturing and clinical trial readiness, is critical, with incentives needed for both non-profit models and private sector engagement (24).

Current global political polarization, nationalism, and denial among parts of the scientific and civil society pose significant barriers to success. The political commitments of groups such as the G7 and G20 are an important starting point, but they must be deepened. The WHO pandemic agreement, under discussion for a long time and finally adopted by the World Health Assembly on 20 May 2025, focuses on equity, collaboration, and innovation as its core principles. However, it needs to consider that governments are first and foremost accountable to the wellbeing of their own populations (25–27). This pandemic agreement aligns with most of the findings and recommendations highlighted in this study, including the following: equity and solidarity during pandemics; timely sharing of pathogen samples and equitable access to resulting health products; technology transfer to benefit LMICs; strengthening of health, regulatory, and surveillance systems; promotion of research and development and local production; and the creation of a logistics network to ensure equitable access to pandemic vaccines. Although legally binding, this historic achievement primarily serves as a blueprint with limited enforceability; until its full ratification by all Member States, its obligations remain voluntary.

Complementary to technological innovations and pandemic policy changes, population readiness must also be addressed. Following the COVID-19 pandemic, rising misinformation, vaccine hesitancy, and trust erosion are undermining immunization programs and vaccine coverage globally (28, 29), especially affecting the most fragile, vulnerable, and high-risk groups (30). Indeed, misinformation has been ranked by the World Economic Forum as the most severe short-term global risk (31), as it generates skepticism about the benefit and safety of vaccines. It should be countered through targeted public communication and education strategies included in pandemic preparedness programs, and the CEPI may consider engaging in this effort to ensure not only the development but also the use of effective tools.

Several core recommendations emerged from this study, proposed by the experts from HICs and LMICs, with a focus on the following priorities:

- Expand, reinforce, and sustainably fund current outbreak surveillance systems in both HICs and LMICs.
- Establish libraries of vaccine prototypes through global collaboration and ensure open access.
- Clarify the responsibilities of different governmental and non-governmental stakeholders in the 100DM, and establish unanimous criteria for the definition of “Day Zero” and the subsequent “reaction phase.”
- Ensure the establishment of “warm” clinical trial sites, increased regulatory maturity, and a functional pharmacovigilance system in LMICs, especially where regionalized manufacturing is planned, prior to or in parallel with setting up manufacturing infrastructure.
- Ensure operational functionality of any new manufacturing site by selecting a platform that can also be used in interpandemic cycles.
- Incentivization of private–public partnerships and multinational treaties specifically designed for pandemic vaccines.
- Address misinformation and increase the visibility of the Mission, especially involving governments and the general population, which should be incorporated into the scope of the CEPI’s initiative.

The strength of our survey lies in the inclusion of stakeholders from both HICs and LMICs, with a high level of expertise, belonging to key sectors involved in vaccine development, access, and policy making. Furthermore, because of the anonymity of the survey, the experts were able to express their opinions without restrictions and biases. The high response rate of 73% among scientists reflects the strong interest of global experts in pandemic preparedness and their willingness to contribute.

An important limitation of the survey is the lack of CMC experts. Secondly, the participation of the experts in LMICs and HICs was not evenly balanced, nearly one-third of the respondents were from LMICs. Lastly, the survey lacked questions related to engaging the public in pandemic preparedness.

In conclusion, the 100DM is an absolutely critical tool for pandemic readiness, but it needs to be better communicated and should focus on meaningful early wins to ensure credibility and secure sustained financing. It also serves as a reminder to all, as Larry Brilliant observed: “Outbreaks are inevitable (the nature part), but pandemics are optional (the human part)” (32).

## Data Availability

The raw data supporting the conclusions of this article will be made available by the authors, without undue reservation.
