# Supplementary material for: Pandemic preparedness and response: a survey among experts from high- and low-middle-income countries about the “100 Days Mission”
Source: Front Public Health. 2025 Sep 5;13:1617066. doi: 10.3389/fpubh.2025.1617066 (PMC12446282; doi:10.3389/fpubh.2025.1617066)
Supplement: Supplementary file 1 [file Data_Sheet_1.PDF]

frontiers

Supplementary Material

1 Copy of the study questionnaire.

Obstacles for the realisation of the "100 Day Mission" in the context of pandemic preparedness: a qualitative study among Key Opinion Leaders from both High and Low-Middle Income Countries

- You participation in this survey is voluntary.
- This survey will be recorded for the purpose of transcribing and reviewing the answers.
- Your responses will be confidential and anonymous.
- The survey's answers will be stored initially with Jotform.com in a password protected electronic format and after the completion of the final thesis all collected questionnaires will be deleted.
- Individual responses will be aggregated for analysis and thesis compilation.
- None of the responses will be reported individually but stratified based on field of expertise and geographical location.
- The results of this study will be used only for the purposed of completing the Master program.

Please select your choice: clicking on "Agree" indicated that you have read the above information and voluntarily agree to participate. *

○ Agree

○ Disagree

Please select your field of expertise *

- ◯ Preclinical Research
- ◯ Manufacturing
- ◯ Clinical Development
- ◯ Regulatory Affairs
- ◯ Public Health
- ◯ Governmental institution
- ◯ Non-Governmental Organizations
- ◯ National Immunization Technical Advisory Group
- ◯ Funding institution
- ◯ Other

Number of years working in the field *

Country where you work *

"Vaccine should be ready for initial authorisation and manufacturing at scale within 100 days of recognition of a pandemic pathogen, when appropriate." This is the definition given by CEPI to summarize the "100 Days Mission" concept. Are you confident that this ambitious goal will become the new default in pandemic preparedness in the future?

- ◯ Very confident
- ◯ Confident
- ◯ Neutral
- ◯ Sceptical
- ◯ Very sceptical

In a World where outbreaks are unavoidable and new pathogens are emerging (while not every new pathogen will emerge into an outbreak of public health relevance or even a pandemic), what would be your definition of "day 0"?

Who should ultimately take the decision to move forward to "day 1", starting the development of a vaccine for a potential treat?

- ◯ The WHO
- ◯ Regional organizations (CDC, eCDC, Africa CDC, PAHO, etc.)
- ◯ Locally the Governments with the guidance of local experts in surveillance
- ◯ Local manufacturers
- ◯ Other

How would you judge the overall level of pandemic preparedness of Low-Middle Income Countries (LMICs) currently compared to the situation prior to the Covid-19 pandemic?

- ◯ Improved
- ◯ Slightly improved
- ◯ Same as before
- ◯ Slightly worsened
- ◯ Worsened

How do you rate the difficulty level in the implementation of the main categories of innovations in pre-pandemic preparedness defined by CEPI.

	Very difficult	Moderately difficult	Difficult	Slightly difficult	Not difficult at all	Comments
Creation of libraries of vaccine prototypes for representative pathogens across multiple virus families of pandemic potential	◯	◯	◯	◯	◯	
Establishment of a sustained "warm base" global clinical trial and laboratory network	◯	◯	◯	◯	◯	
Identification of early biological markers that predict vaccine clinical protection ("correlate of protection") when conventional vaccine efficacy trials are not feasible	◯	◯	◯	◯	◯	
Creation of a sustained "warm base" global biomanufacturing capacity	◯	◯	◯	◯	◯	
Establishment of a reliable, sophisticated, active and continuous global disease surveillance system	◯	◯	◯	◯	◯	

It is important that both manufacturing facilities and clinical trial networks are kept "warm". How practically achievable can this goal be?

- ◯ Very achievable
- ◯ Achievable
- ◯ Unachievable
- ◯ Completely unachievable

Could you briefly name the one most critical issue connected to the implementation of each category of innovation?

	Issue
Creation of libraries of vaccine prototypes	
Establishment of a global clinical trial and laboratory network	
Identification of early biological markers that predict vaccine clinical protection	
Creation of a global biomanufacturing capacity	
Establishment of an active and continuous global disease surveillance system	

Beside the issues with the "100 Days Mission" innovations, how do you rate the following factors in terms of challenges in pre-pandemic preparedness, in reaction phase, and in roll-out and reviews during a potential pandemic, especially in LMICs?

	Very important	Moderately important	Important	Slightly important	Not important at all	Comments
Lack of governance and political will	◯	◯	◯	◯	◯	
Insufficient long-term investments and financing at risk for products and activities in pandemic preparedness	◯	◯	◯	◯	◯	
Rolling reviews of Regulatory Authorities with low resources and capabilities	◯	◯	◯	◯	◯	
Lack of Pharmacovigilance systems	◯	◯	◯	◯	◯	
Lack of dedicated workforce	◯	◯	◯	◯	◯	
Inadequate and insufficient systems of shipping and delivery vaccines to the population at risk globally	◯	◯	◯	◯	◯	
Logistical difficulties in terms of supply chain for vaccines, raw materials and consumables during outbreaks	◯	◯	◯	◯	◯	
Lack of global and local effective collaborations among the stakeholders during outbreaks	◯	◯	◯	◯	◯	
Competing concepts, prioritisation of national over international perspectives, and misalignment on roles and responsibilities in the pandemic preparedness ecosystem	◯	◯	◯	◯	◯	

In your opinion, are there any other critical factors that have not been mentioned above?

◯ Yes

◯ No

If yes, please specify at least one critical factor (please do not hesitate to provide critical feedback)

The aim of the “100 Days Mission” ultimately is to have a vaccine ready to be administered at day 100 initially to the population at risk globally. In the Covid-19 pandemic there were clear inequalities between countries. How do you think it will be possible to overcome these inequalities, especially between High Income Countries (HICs) and LMICs, in order to have equal access as part of the “100 Days Mission”?

Do you think that the “100 Days Mission” concept - as proposed currently - will be suitable to overcome existing challenges in terms of equitable access to vaccines for populations in need, considering the context of HICs and LMICs?

- ○ Yes
- ○ No

If no, why?

Is there anything that you would like to add and/or to comment about the mission proposed by CEPI and its practicality?
